# Cortical folding complexity and attentional impulsivity in chronic cocaine users: an MRI study

**DOI:** 10.1192/j.eurpsy.2023.720

**Published:** 2023-07-19

**Authors:** N. Trevisan, F. Di Camillo, G. Cattarinussi, N. Ghiotto, M. Sala, F. Sambataro

**Affiliations:** ^1^Department of Neuroscience (DNS); ^2^Padova Neuroscience Center, University of Padova, Padua, Italy; ^3^Department of Psychological Medicine, Institute of Psychitry, Psychology & Neuroscience, King’s College London, London, United Kingdom; ^4^Department of pathophysiology and Transplantation, University of Milan, Milan, Italy

## Abstract

**Introduction:**

Cocaine use is a worldwide health problem with psychiatric, somatic, and socioeconomic complications, being the second most widely used illicit drug in the world. Despite several structural neuroimaging studies, the alterations in cortical morphology associated with cocaine use and addiction are still poorly understood. Interestingly, findings from human studies and animal models examined how impulsivity is a risk factor for the emergence of substance use disorders.

**Objectives:**

In this study we aimed at investigating the complexity of cortical folding (CCF) in patients with cocaine addiction using fractal dimension (FD). Since the frontal, parietal, temporal and insular cortices have been shown to play an important role in decision making and impulsivity, we hypothesized that CCF in the brain of patients would be altered in these regions. Furthermore, we predicted the association between CCF changes and 1) the duration of cocaine use for its widespread neurotoxic effects and 2) impulsivity characteristics in those regions implicated in the predisposition to addiction.

**Methods:**

We compared the CCF between patients with cocaine addiction (n=52) and controls (n=36) and correlated it with characteristics of addiction and impulsivity. Demographic data, history, and current substance use were collected. Furthermore, the Barratt Impulsivity Scale (BIS-11) was administered.

**Results:**

**Table:** 

	**Patients with cocaine addiction (N=52)**	**Healthy controls** **(N=36)**	**χ2 or t**	***p***
Age (M±SD, years)	31.3 ± 6.51	30.1 ± 7.62	0.795	0.429
Education (M±SD, years)	10.9 ± 2.9	13.2 ± 3.53	-3.276	0.002
Total intracranial volume (M±SD, μl)	1442.7 ± 104.6	1460.4 ± 98.31	-0.796	0.428
BIS Total score (M±SD)	61.1 ± 14.6	40.2 ± 10.4	6.52	<0.001
BIS Attentive score (M±SD)	17.1 ± 5.23	11.6 ± 5.23	4.79	<0.001
BIS Motor score (M±SD)	18.4 ± 7.79	13.3 ± 5.72	2.97	0.004
BIS NonPlanning score (M±SD)	25.6 ± 6.82	15.3 ± 5.29	6.70	<0.001
Duration of cocaine use (M±SD, years)	10.8 ± 6.4			
Age of onset of cocaine use (M±SD, years)	20.7 ± 4.99

We found that patients with cocaine addiction had greater attentional impulsivity compared to HC. In addition, they showed reduced CCF in a cluster that encompassed the left insula and the supramarginal gyrus (SMG) and in one in the left medial orbitofrontal cortex. Moreover, the CCF in the left medial orbitofrontal cortex was correlated with the age of onset of cocaine addiction and with attentional impulsivity.

**Image:**

**Figure:**
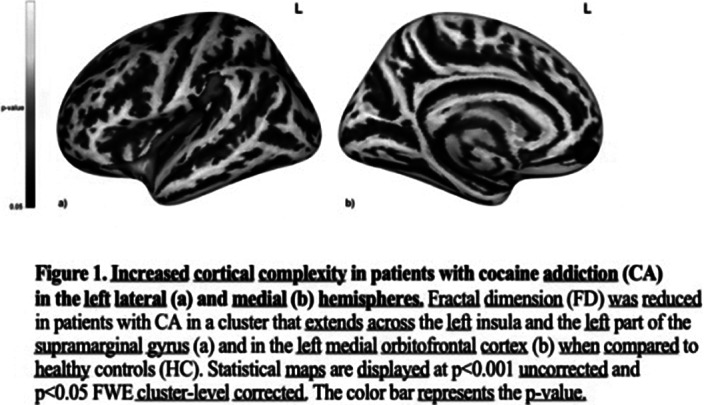

**Image 2:**

**Figure:**
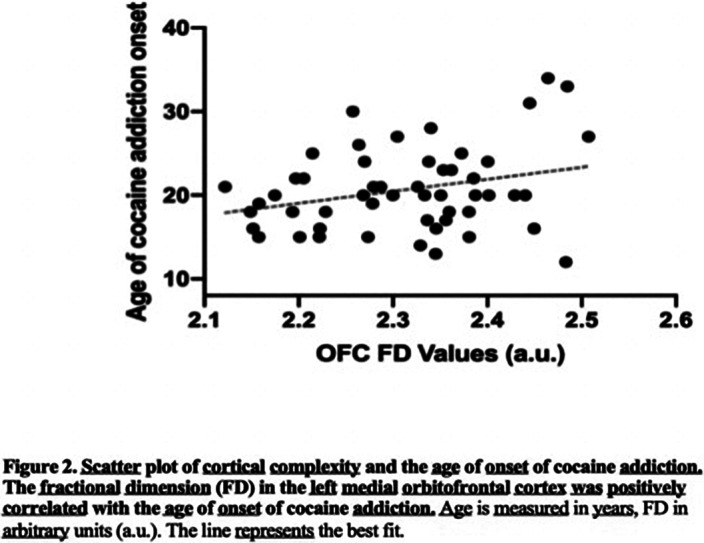

**Conclusions:**

Overall, our findings suggest that chronic cocaine use is associated with changes in the cortical surface in the fronto-parieto-limbic regions that underlie emotional and attentional regulation, and these changes are associated with prolonged cocaine use. Future longitudinal studies are warranted to unveil the association of these changes with the diathesis for the disorder or with the chronic use of this substance.

**Disclosure of Interest:**

None Declared

